# Establishing Consensus on the Breakthrough Pain Assessment Questionnaire-Self Report (BTPAQ-SR) for Typically Developing Children and Young People (8–25 yrs) with Life-Limiting and Life-Threatening Conditions: An International e-Delphi Study of Expert Healthcare Professionals

**DOI:** 10.3390/children12121627

**Published:** 2025-11-30

**Authors:** Christina Liossi, Georgia Turner, Anna-Karenia Anderson, Emily Harrop, Simon Bailey, Margaret Johnson, Christine Mott, Daniel Eric Schoth, James Hayden, Dilini Rajapakse, Kate Renton, Bernie Carter

**Affiliations:** 1 Pain Research Laboratory, School of Psychology, University of Southampton, Highfield, Southampton SO17 1BJ, UK; g.e.m.turner@soton.ac.uk (G.T.); ottersriverbank@natsirt.co.uk (M.J.); d.e.schoth@soton.ac.uk (D.E.S.); 2 Psychological Services, Great Ormond Street Hospital for Children NHS Foundation Trust, Great Ormond Street, London WC1N 3JH, UK; 3 Royal Marsden Hospital, Sutton SM2 5PT, UK; annakarenia.anderson@nhs.net; 4 Helen & Douglas House Hospices, Oxford OX4 1RW, UK; eharrop@helenanddouglas.org.uk; 5 Great North Children’s Hospital, Queen Victoria Road, Newcastle upon Tyne NE1 4LP, UK; simon.bailey@newcastle.ac.uk; 6 Birmingham Children’s Hospital and Acorns Children’s Hospice, 103 Oak Tree Lane, Selly Oak, Birmingham B29 6HZ, UK; christine.mott2@nhs.net; 7 Alder Hey Children’s NHS Foundation Trust, Eaton Road, Liverpool L12 2AP, UK; james.hayden@alderhey.nhs.uk; 8 Louis Dundas Centre for Oncology Outreach and Children’s Palliative Care, Great Ormond Street Hospital for Children NHS Foundation Trust, London WC1N 3JH, UK; dilini.rajapakse@gosh.nhs.uk; 9 University Hospital Southampton NHS Foundation Trust, Southampton SO16 6YD, UK; kate.renton@uhs.nhs.uk; 10 Faculty of Health, Social Care and Medicine, Edge Hill University, St Helens Rd, Ormskirk L39 4QP, UK; bernie.carter@edgehill.ac.uk

**Keywords:** breakthrough pain, pain assessment, life-limiting conditions, life-threatening conditions, paediatric pain, palliative care, cancer

## Abstract

**Highlights:**

**What are the main findings?**

- The diagnosis of breakthrough pain (BTP) remains controversial.
- Preliminary evidence supports the construct validity of the newly developed BTPAQ-SR.

**What is the implication of the main findings?**

- There is an urgent clinical need for conceptual clarity around breakthrough pain and the development and adoption of a standardized, consensus-based assessment tool in paediatrics.

**Abstract:**

Background/Objectives: Breakthrough pain (BTP) is commonly experienced by children and young people with life-limiting and life-threatening conditions. While over 50 tools exist for the assessment of breakthrough pain in adults, there is currently no standardised measure designed for use in paediatrics. To address this gap, the multi-phase BEACON clinical trial aims to develop the Breakthrough Pain Assessment Questionnaire (BTPAQ) for use with children and young people with life-limiting and life-threatening conditions aged 3 months to 25 years. The goal of the current study was to refine the self-report version (BTPAQ-SR) of the questionnaire through an international, sequential, electronic-Delphi process. Methods: Healthcare professionals with at least three years of clinical experience working with children and young people with life-limiting and life-threatening conditions were invited to complete an anonymous online survey. The alpha version of the BTPAQ-SR was developed from systematic reviews, qualitative interviews, and the BEACON Steering Group. It had a diagnostic algorithm (Part A) and 18 items (Part B); however, items that included multiple descriptors or options were separated and presented individually, resulting in 49 survey items being presented to participants. Participants rated the importance of all survey items to assess breakthrough pain and the frequency of presentation for a subset of 37 items. Results: Fifty-three healthcare professionals from nine different countries were recruited, the majority of whom were physicians or nurses. Of the 49 survey items, 46 (93.8%) reached the ≥70% consensus threshold for importance, and 31 (83.8%) of 37 reached consensus for frequency. In total, 42 survey items reached consensus for both importance and frequency. Conclusions: The findings from this study support the clinical need for the BTPAQ-SR, confirm its conceptual foundation, and justify its continued development. Next steps include cognitive interviews with children and young people and introduction to clinical care to assess the psychometric properties of the BTPAQ-SR, including its clinical utility, reliability, and validity.

## 1. Introduction

Patient-Centred Outcome Measures, including patient-reported outcome measures (PROMs), patient-reported experience measures, goal-based outcomes, and proxy measures, are essential tools for assessing patient health outcomes. In palliative care, PROMs support diagnosis, assessment, symptom management and communication between the patient and healthcare provider [1,2]. They also contribute to service evaluation and research by providing insight into care quality and the impact of interventions [3,4].

PROMs for children and young people with life-limiting and life-threatening conditions (LLC/LTCs) present significant challenges, primarily due to the developmental differences among this population and the heterogenous nature of the conditions [5,6,7]. Pain is a particularly critical and complex domain within PROMs, due to its subjective nature, developmental variations in expression, and the influence of underlying conditions [8,9]. Consequently, current knowledge in developing suitable pain assessment tools remains underdeveloped leading to further challenges in optimal pain management in the paediatric population [10]. This is especially challenging for breakthrough pain (BTP). Despite a high prevalence rate among children and young people with LLC/LTCs [11,12,13], BTP diagnosis and management in this population is hindered due to the lack of agreed upon definition and characterisation of BTP [14,15].

A systematic review of BTP measures by our group [16] identified over 50 tools available for BTP assessment of adults patients, including two diagnostic algorithms developed to aid in BTP diagnosis: the Breakthrough Cancer Pain Algorithm [17,18], and the Davies Algorithm [19,20]. However, there is currently no standardised measure specifically designed for use in the paediatric patient population [16].

To address this gap, following COnsensus-based Standards for the selection of health Measurement INstruments (COSMIN) [21,22,23] and expert guidelines (FDA; U.S. Department of Health and Human Services: Food and Drug Administration [24], ISPOR; Matza, Patrick [25], Initiative on Methods, Measurement and Pain Assessment in Clinical Trials (IMMPACT); Turk, Dworkin [26]) for developing and evaluating validated patient-reported outcome measures, the multi-phase BEACON clinical trial aims to develop the Breakthrough Pain Assessment Questionnaire for use with children and young people with LLC/LTCs, aged 3 months to 25 years. Two forms of the questionnaire will be developed: a self-report form (BTPAQ-SR) and a form for pre- or non-verbal children and young people to be completed by a parent, carer or healthcare professional. A pre-development phase established the need and support for a validated measure to diagnose, characterise, and assess BTP in patients aged 3 months to 25 years. This was achieved through systematic reviews [27,28,29], qualitative interviews [30], Patient and Public Involvement (PPI) input, and expert consensus (UK NIHR Clinical Research Network Children, Pain & Palliative Care Clinical Research Group).

The primary goal of the current study was to refine the BTPAQ-SR through an international, sequential, electronic Delphi (e-Delphi) process, recruiting healthcare professionals with at least three years of clinical experience working with children and young people with life-limiting/life-threatening conditions. The e-Delphi method is especially suitable when a subject lacks empirical evidence, and has been widely applied in paediatric healthcare research, particularly PROM development [31,32], and paediatric palliative care outcome development [33,34]. Given the lack of agreement surrounding BTP diagnosis, employing the Delphi methodology to obtain expert opinion and consensus of the BTPAQ-SR was considered essential. The use of an e-Delphi format also facilitated international panel representation, while allowing flexibility and anonymity in expert feedback [35].

The alpha version of BTPAQ-SR developed by BEACON team members (C.L., A-K.A., E.H., S.B., M.J.P., C.M., D.R., K.R., B.C.), consisted of 19 items, divided into two sections (A and B): Section A presents a diagnostic algorithm intended for use by healthcare professionals to diagnose breakthrough pain (BTP); Section B comprises 18 items designed to assess various aspects of BTP. These include a body map (Item 1), triggers of BTP (Items 2 and 3), intensity (Items 4 and 5), onset (Items 6 and 7), frequency (Item 8), duration (Item 9), pain descriptors (Item 10), and free-text responses regarding factors that alleviate or exacerbate BTP (Items 11 and 12). Emotional aspects are documented in Items 13 to 16, followed by the impact of BTP on activities of daily living (Item 17), and a final free-text box for any additional relevant information (Item 18).

The aim of this modified e-Delphi study was to achieve expert consensus of questionnaire items for inclusion in the BTPAQ-SR for typically developing children and young people aged 8 to 25 years with LLC/LTCs, and to obtain feedback on item wording and response options.

## 2. Materials and Methods

Ethics approval was granted by the University of Southampton Research Ethics Committee (ref: #90597). The design and reporting of the e-Delphi adhered to the CREDES (Guidance on Conducting and Reporting Delphi Studies; [36]), and the study protocol was pre-registered with Open Science Framework (https://osf.io/6jn8y/, on 3 July 2024). No significant protocol violations occurred. The wider programme of research that this e-Delphi study will inform is registered and listed on the ClinicalTrials.gov ID, with study registration number NCT06790719 (submitted on 17 January 2025).

### 2.1. Design

A modified e-Delphi methodology was adopted to evaluate the content validity of the initial item pool of the alpha version of the BTPAQ-SR. The term ‘modified Delphi’ is used variably in the literature and at times inconsistently [37,38]. In this study, it refers to an e-Delphi conducted entirely online with no participant meetings, in which the maximum number of rounds was defined a priori. Open-ended questions in each round allowed participants to provide additional comments, combining structured consensus methods with opportunities for richer qualitative input [35].

The BEACON steering group developed the Delphi survey, which was subsequently reviewed by three healthcare professionals experienced in Delphi methodology (from six invited). These experts suggested improvements to the formatting of the Delphi survey and provided linguistic recommendations to enhance the clarity of the introductory text. All suggestions were reviewed by the team and implemented as appropriate. Before finalising, the survey was pre-tested with four different healthcare professionals. Completion time averaged approximately 15 min.

**Figure 1:**
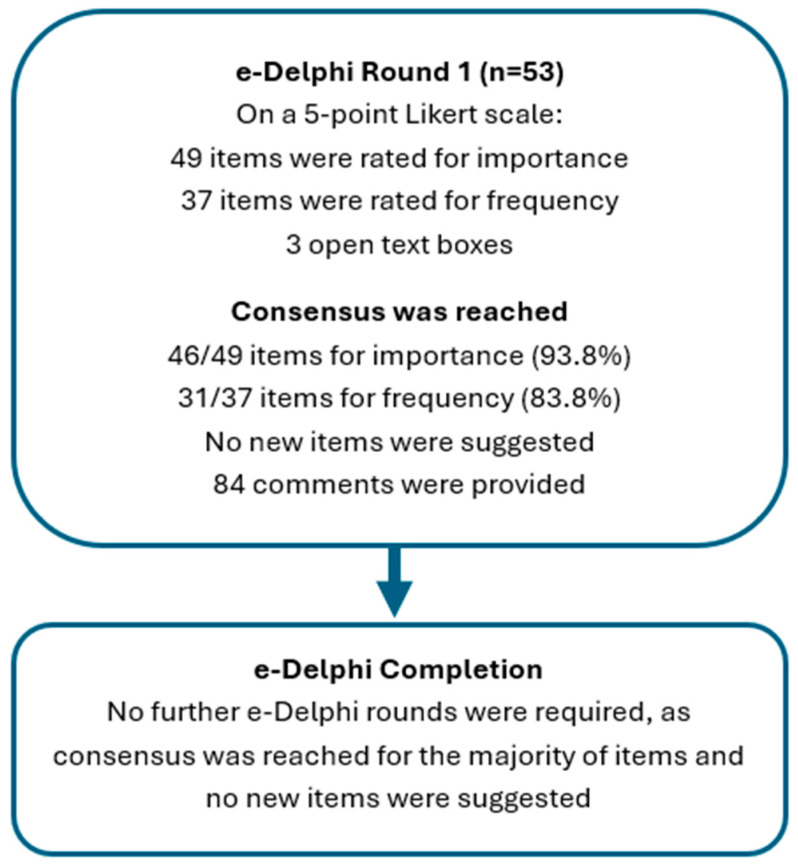
Flow diagram illustrating the one round of the e-Delphi study where consensus was achieved.

Given the high level of consensus achieved in Round 1, the absence of items meeting non-consensus criteria, and the lack of suggestions for additional items, further e-Delphi rounds were not necessary, contrary to what we had initially anticipated (see Figure 1 for the e-Delphi process).

### 2.2. Expert Panel

Healthcare professionals were eligible for inclusion in this study if they had at least three years of clinical experience working with children and young people with LLCs or LTCs. Exclusion criteria were (i) lack of sufficient clinical experience, (ii) identified conflicts of interest, and (iii) inability to commit enough time to complete the e-Delphi process.

A purposive sampling strategy was used to recruit a panel of national and international experts in paediatric breakthrough pain. Experts were identified through national and international organisations, including the Association of Paediatric Palliative Medicine (APPM), International Society of Paediatric Oncology (SIOP), International Children’s Palliative Care Network (ICPCN), and the Children’s Cancer and Leukaemia Group (CCLG). Members of the BEACON clinical trial steering group also disseminated invitations within their professional networks (Pediatric Pain List). To maximise reach, the study was promoted via social media (i.e., X), and professional platforms (i.e., LinkedIn), and a snowball sampling strategy [39] was used to encourage participants to forward the survey to relevant colleagues. The aim was to have 100–200 participants in each e-Delphi round, regardless of whether they had participated in a previous round.

### 2.3. Data Collection and e-Delphi Survey

The e-Delphi survey was administered using Qualtrics [40]. Participants anonymity was assured. No incentives were offered. Participant information sheets were provided and consent gained by ticking a consent box at the start of the survey. Participants then provided core demographic and clinical experience data (gender, ethnic background, profession, primary role, country of practice, years of experience in pain management, number of patients aged 8–25 years with LLC/LTCs cared for clinically per year, and percentage of these who have BTP).

The alpha version of the BTPAQ-SR had a diagnostic algorithm (Part A) and 18 items (Part B); however, items that included multiple descriptors or options were separated and presented individually, resulting in 49 survey items being presented to participants. Survey items were evaluated for importance and frequency of presentation. Participants were asked to rate importance for all 49 survey items and frequency for a subset of 37 survey items deemed appropriate for that metric.

For Part A, participants were asked *“How important is the algorithm in screening for breakthrough pain”.* For Part B, participants were asked *“How important is this item in assessing breakthrough pain?”* and *“How frequently do your patients with breakthrough pain experience this?”* Participants rated importance using a 5-point Likert scale *(Not at all*, *Somewhat*, *Important*, *Very important*, and *Extremely important).* Frequency was rated on a separate 5-point scale *(Never/Almost Never*, *Rarely*, *Sometimes*, *Often*, and *Always/Almost Always).* Additionally, open-ended text boxes were included at four points throughout the survey to gather qualitative feedback.

### 2.4. Consensus Criteria

Consensus criteria were defined a priori and reported in the published protocol (https://osf.io/6jn8y/, on 3 July 2024). Following guidelines from current Delphi literature [36,41,42] consensus was deemed to be achieved if ≥70% of participants rated an item as “Important” or higher (3–5) for importance, and “Sometimes” or higher (3–5) for frequency. An IQR of ≤1 was used as an additional indicator of consensus. Conversely, if ≥70% of participants rated an item as “Not at all important” or “Somewhat important” (1–2) and/or “Never/Rarely” (1–2) for frequency, the item was considered for exclusion.

### 2.5. Data Analysis

Quantitative data were analysed with IBM SPSS Statistics 27. Descriptive statistics (number, percentage) were used to summarise participant demographic and clinical experience data and item-level responses. Items were analysed for frequency distribution, mode, and interquartile range (IQR). Data visualisations, including bar charts, were created using SigmaPlot version 15.0 [43].

Qualitative data from the open text boxes were analysed using inductive content analysis [44,45,46]. Responses were exported into NVivo (version 14 [47]) and read repeatedly for familiarisation, then coded by two researchers (G.T., C.L.). Any disagreements were discussed with another member of the research team (B.C.) until consensus was reached.

## 3. Results

### 3.1. Characteristics of the Panel

Round one of the e-Delphi had 53 participants. Of these, two participants were excluded due to having less than 3 years’ experience in pain management, and one participant was excluded due to not providing care for patients with BTP. There was no missing data.

The socio-demographic and clinical experience characteristics of participants are presented in Table 1. Most participants were female (46, 92%) and were primarily British or Irish (30, 60%). The expert panel consisted primarily of physicians (26, 52%) and nurses (21, 42%), with only three allied health professionals (6%). The majority identified their primary role as clinicians (46, 92%). Most panellists were based in the United Kingdom (38, 76%), with the remainder practicing in the USA, Canada, Australia, New Zealand, Sweden, Malawi, and Panama. Participants reported a range in years of clinical experience; 68% had over 10 years of experience in pain management, and most (45, 90%) managed more than 10 patients aged 8–25 years with LLC/LTCs annually. Forty percent (20) of participants indicated that between 25 and 50% of their patients experienced BTP, while 44% (22) reported BTP prevalence in over 50% of patients they care for.

### 3.2. Quantitative Results

Of the 49 survey items, 46 (93.8%) items reached consensus for importance, while 31 (83.8%) out of 37 items reached consensus for frequency. Forty-two items reached consensus for both importance and frequency. No items met the criteria for non-consensus (i.e., ≥70% agreement in the 1–2 Likert response range).

**Figure 2:**
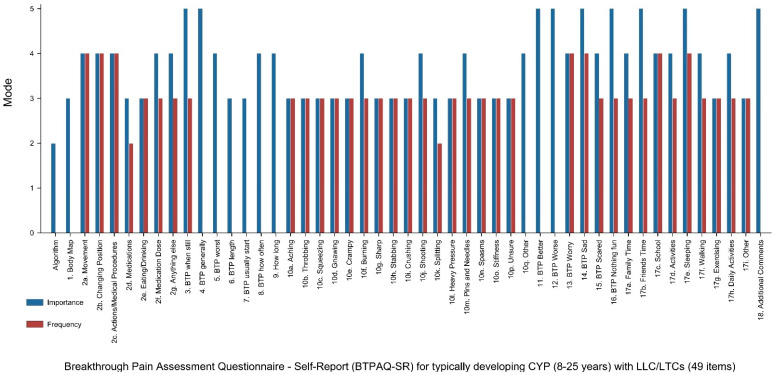
Mode scores for participant rated importance and frequency of all 49 items in the Breakthrough Pain Assessment Questionnaire—Self Report (BTPAQ-SR) for typically developing children and young people (8–25 years) with LLC/LTCs.

An overview of mode scores across items in the BTPAQ-SR for both importance and frequency ratings is presented in Figure 2. The mode and IQR for each item assessed are presented in Appendix A, reflecting the level of consensus on both importance and frequency. Individual total response to each survey item, along with grouped consensus across the Likert scale (3–5) can be found in Appendix B.

The diagnostic algorithm in Section A did not meet the importance threshold, with only 54% of experts rating it between 3 and 5. Similarly, two items failed to reach consensus on both importance and frequency: (a) Item 2d: “The following bring on my breakthrough pain: Medications” (Importance consensus: 68%; Frequency: 32%), (b) Item 10k: “My breakthrough pain feels like: Splitting” (Importance: 64%; Frequency: 34%)

An additional four items met consensus for importance, but not for frequency: (a) Item 2f: “Needing another dose of my normal pain medication” (Importance: 80%; Frequency: 68%), (b) Item 10d: “Gnawing” (Importance: 80%; Frequency: 60%), (c) Item 10i: “Crushing” (Importance: 80%; Frequency: 56%), (d) Item 10l: “Heavy pressure” (Importance: 80%; Frequency: 54%).

### 3.3. Qualitative Results

Overall, 80% (n = 40) of the expert panel provided at least one response to the open-ended questions; 84 comments were documented in total. Fifteen comments addressed concerns about self-report measures being unsuitable for pre- and non-verbal children and young people, as well as concerns about survey length and repetitiveness. These comments were excluded from the analysis as they pertained to well-established and widely acknowledged limitations. It is important to note that (a) the BTPAQ-SR reviewed within the e-Delphi survey was specifically designed for typically developing, verbal young people and (b) the e-Delphi survey, due to the nature of the methodology, was necessarily long and repetitive unlike the actual questionnaire.

Content analysis of the remaining open-text responses yielded three overarching themes: A Controversial Diagnostic Algorithm: Challenges and Considerations, BTPAQ-SR: A Valuable Assessment Tool in Need of Refinement, and Key Considerations in the Assessment of Breakthrough Pain and the Role of the BTPAQ-SR (see Table 2).

Although four participants provided short comments such as “clear” and “easy to use” to “not sure it’s hugely useful”, which provided limited information beyond a general view of the BTPAQ-SR, most other responses offered deeper insights. Quotations are attributed via participant number (e.g., P1, P2), role (e.g., physician, nurse) and years of pain management experience (PME).

### 3.4. A Controversial Diagnostic Algorithm: Challenges and Considerations

Twenty-nine participants provided feedback for the algorithm. Eight participants welcomed the inclusion of a diagnostic algorithm in the questionnaire, typically describing the algorithm as a:

“*Very clear clinical decision-making tool*”.

(P39: Nurse, >10 yrs’ PME)

Two participants specifically praised its educational value for less experienced healthcare professionals at the beginning of their healthcare career or transitioning from a different area of healthcare where breakthrough pain is not as prevalent.

One area where views were divided was whether a patient’s background pain must be well-controlled before being diagnosed with breakthrough pain. Some participants (n = 3) appreciated the algorithm’s clarity in distinguishing BTP from background pain.

“*Helpful for those who cannot differentiate between BTP and background pain*”.

(P5: Physician, >10 yrs’ PME)

“*It helped me clarify that you were specifically thinking breakthrough above medically managed background pain. I tend to lump breakthrough and acute, episodic pain together in my mental model*”.

(P8: Physician, >10 yrs’ PME)

While for others (n = 6), this distinction was problematic. One proposed that some patients without background pain may still experience BTP, and another mentioned the following:

“*If the background pain is not well controlled, it may be because you are still titrating their background regimen to effective dosing, so I am not clear why you would say no breakthrough pain for this*”.

(P9: Physician, 3–5 yrs’ PME)

Another element of the diagnostic algorithm discussed by six participants was the ‘12 h’ classification within the algorithm. They noted that pain patterns can vary depending on the condition, making this criterion “*crude*” and “*potentially confusing*”.

“*We would not base it on 12 h a day of pain but on regular intervals/ periods etc throughout the day/nights*”.

(P1: Nurse, >10 yrs’ PME)

Similarly, one physician found the use of ‘*brief’* in relation to BTP to be “*vague*”, and another mentioned that pain increases may be “*brief or prolonged*”.

Some suggestions for improvement included revising initial instructions and clarifying terminology:

“*It would be helpful to be more explicit in the initial paragraph above the algorithm that even though we may be saying ‘no breakthrough pain’ we are not denying the presence of pain. It is just not termed ‘breakthrough pain’*”.

(P18: Physician, 6–10 yrs’ PME)

### 3.5. BTPAQ-SR: A Valuable Assessment Tool in Need of Refinement

Twenty-six participants provided comments regarding Part B of the BTPAQ-SR. Three participants praised its clarity and structure, noting that it “*reads well*” and was “*easy to follow and well structured*”, with one noting

“*I liked the response format*”.

(P23: Nurse, >10 yrs’ PME)

Others felt that it was “*very useful*”, and “*beneficial for many clinical settings*”. One participant highlighted the value of the tool for enhancing personalised care planning:

“*I find the wording useful as it allows for children and young people to elaborate…which can help in terms of targeting pain management better*”.

(P19: Physician, 3–5 yrs’ PME)

There was also support for the inclusion of items assessing the impact of breakthrough pain on daily activities, emotional well-being, and quality of life. Five participants appreciated the inclusion of items on emotional and functional impact, with one noting that it

“*Provides a way of focusing assessment to the individual’s needs*”.

(P5: Physician, >10 yrs’ PME)

Three participants noted the importance of distinguishing BTP from other pain types (e.g., procedural pain).

“*It will be important to separate out incident pain, procedural pain and pain on specific activities from unprovoked breakthrough pain*”.

(P007, physician, over 10 years’ PME)

Fourteen participants commented on the pain descriptors, with five highlighting the importance of meaning behind the descriptors, noting that some terms (e.g., splitting, crushing, gnawing) might not be understood by children and young people:

“*Some words are common (sharp, shooting, aching) and other words don’t necessarily equate to pain for some people…especially if they have other types of symptoms*”.

(P5: Physician, >10 yrs’ PME)

“*I am not sure the children we see have the vocabulary to differentiate between crushing/splitting/gnawing*”.

(P23: Nurse, >10 yrs’ PME)

One participant emphasised that the descriptors must be meaningful and interpretable for both the clinician and the child, noting that ‘splitting’ caused them some confusion:

“*The words used need to mean something to both clinician and patient—I struggled to understand what was meant by some words e.g., splitting so listening to the child is actually really important*”.

(P32: Physician, >10 yrs’ PME)

Five participants also raised concerns about activity-based items not being applicable to all patients, especially those with life-limiting conditions:

“*For many of the kids…they do not go to school, are not seeing friends, are not able to walk…This may upset them to reflect on all the things they are not doing*”.

(P13: Physician, 3–5 yrs’ PME)

This participant went on to propose alternative language, such as

“*‘moving around’ instead of walking” or ‘doing things I enjoy’ instead of sport or exercise*”.

(P13: Physician, 3–5 yrs’ PME)

Ten participants provided feedback on the structure and response format of the BTPAQ-SR. One found early questions to be “*strangely worded*” and reported selecting “*not important*” due to confusion, and preferred the phrasing of later questions:

“*The wording in this second part… feels more like you can assess it with specific statements*”.

(P9: Physician, 3–5 yrs’ PME)

One physician, whose first language was not English, commented on the phrasing of “*bring on*,” noting it was “*not a very familiar way of expressing*” the intended meaning and suggesting it could be simplified.

An experienced clinical nurse acknowledged the usefulness of a 0–10 rating scale and suggested other alternatives suitable to children, noting the need to consider children’s understanding of the frequency descriptors:

“*I don’t think using words such as occasionally, sometimes is always helpful as kids don’t always understand what that means*”.

(P47: Nurse, >10 yrs’ PME)

### 3.6. Key Considerations in the Assessment of Breakthrough Pain and the Role of the BTPAQ-SR

Three participants acknowledged the complexity of BTP assessment. Others focused on aspects of BTP. One participant noted that the tool “*highlights the importance of the definition of breakthrough pain*”, while another recognised that “… *diagnosis of BTP is important as a means of planning management*”, and a nurse stated

“*I think it is brilliant that this [BTP] is being addressed… Any steps to reduce this can only be a good thing. Thank you*”.

(P23: Nurse, >10 yrs’ PME)

One participant while recognising the value in the BTPAQ-SR, expressed their concern about using assessment measures in general and warned against over-reliance on tools at the expense of individualised care:

“*I think we are not always good at assessing breakthrough pain objectively and a tool to help this would be valuable. I’ve been doing this a long time however, and am really wary of people not listening in a meaningful way to patients and substituting this with tick boxes and scores to direct decision making and potentially not respond to what the patient actually needs*”.

(P32: Physician, >10 yrs’ PME)

## 4. Discussion

A one-round e-Delphi survey was conducted to assess the content validity of the items in the alpha version of the BTPAQ-SR. The aim was to determine expert consensus on the importance and frequency of items related to the diagnosis and assessment of breakthrough pain (BTP) in children and young people and to gather feedback on item wording and response options. Of the 49 survey items presented, 42 reached the pre-established consensus threshold of ≥70%, providing strong support for the tool’s construct validity.

The diagnostic algorithm in Section A did not reach consensus, with 54% of experts rating it important. The current e-Delphi study revealed diverse opinions on two key aspects: the requirement of well-controlled background pain and the distinction between breakthrough, episodic, and end-of-dose pain types. These findings align with known challenges in defining BTP criteria [14,15,16,18,29]. Controlled background pain for the diagnosis of BTP is a recognised criterion [17,19,48], but its interpretation varies significantly across studies. Portenoy and Hagen [48] defined it as “12 h of mild or no pain,” while Webber, Davies [17] used the broader term “most of the time,” a difference that adds to diagnostic ambiguity. The Breakthrough Pain Assessment Tool’s validation study (BAT; [18]) demonstrated limited diagnostic accuracy, identifying only 54% of 65 clinically confirmed BTP cases.

Despite this lack of diagnostic consensus, developing a validated diagnostic tool remains crucial, especially for paediatric populations, where none currently exists [16]. Therefore, the diagnostic algorithm will be retained and further evaluated as part of the psychometric assessment of the BTPAQ-SR. The lack of agreement among some respondents regarding aspects of the definition of breakthrough pain (BTP) highlights the importance of consistent reporting through a standardised, agreed-upon tool. Without such a tool, it would be difficult to ensure consistent assessment, reporting and perhaps management of BTP across services and individual professionals. Achieving sufficient international consensus on this tool provides a strong mandate to meaningfully address this issue. Given the established difficulty in reaching agreement on BTP definitions, the research team’s decision was that conducting a second Delphi round for this item would serve no purpose.

Clinicians responded very positively to the breakthrough pain questionnaire self-report, specifically the version designed for young people to complete. Most items achieved consensus on both importance and frequency, and qualitative comments reflected this strong support. Clinicians valued the structure and clarity the questionnaire brings to breakthrough pain assessment. They noted that nothing comparable currently exists in the literature, making it a valuable addition. The response format was familiar to clinicians and considered appropriate for the target population. Several suggestions for refinement were also provided.

Two items did not reach consensus for either importance or frequency. However, item 2d (“The following bring on my breakthrough pain: Medications”) approached consensus at 68%. After discussion, this item was retained for further consideration with the intention to clarify wording. Item 10k (“My breakthrough pain feels like: Splitting”) did not reach consensus for either importance or frequency. Similarly, Item 10d (“Gnawing”) did not meet consensus for frequency. Both items were provisionally marked for removal and will specifically undergo further evaluation during think-aloud interviews with children and young people experiencing breakthrough pain, those with other medical conditions, and healthy peers to confirm this decision. Research indicates that younger children typically use a limited and concrete vocabulary to describe pain [49,50], with more metaphorical and sensory-rich language emerging later in development, generally from around eight years of age. In the UK, for example, this developmental stage coincides with increased academic demands in the national curriculum for English and mathematics. Expectations at this level include abilities such as deducing, inferring, and interpreting information from multiple sources in a text, as well as grasping mathematical concepts like division, fractions, decimals, and unit conversions. However, adults often underestimate children’s linguistic and cognitive capabilities, shaped in part by their own experiences and assumptions about language use and development. Other descriptors that did not reach consensus for frequency included Item 10i (“Crushing”) and Item 10l (“Heavy Pressure”). Although these descriptors were less frequently seen, they did reach consensus for importance. As their potential relevance to the target population was recognised, the decision was made to retain them at this stage. Importantly, the BTPAQ-SR allows children and young people to provide their own descriptions and includes space throughout for elaboration in their own words; an element seen as important by the e-Delphi participants.

A key strength of this study was its international, multidisciplinary expert panel, which surpasses previous BTP studies that used only national panels (e.g., [17], *n* = 14 UK experts; [51] *n* = 15 Australian experts) and exceeds that of similar Delphi studies on PROMs ([52] *n* = 15; [53] *n* = 30). Although broader geographical representation would improve reliability, time constraints of specialists in this field limited recruitment, a common challenge in Delphi research [54,55]. Similarly, the e-Delphi methodology does not enable real-time interaction or in-depth discussion among participants, but it offers flexibility by allowing those with limited time to contribute at their convenience. Another key strength was that the research adhered to a modified e-Delphi methodology, including protocol development and pre-registration with transparent selection processes, pre-defined consensus thresholds that were more stringent than previous work (e.g., 50% consensus in [56]), and inclusion of qualitative data [35,36].

## 5. Conclusions

This e-Delphi study achieved consensus on 42 of 49 survey items in the alpha version of the BTPAQ-SR. Agreement on the majority of assessment items provided preliminary validation of the tool’s construct validity, format and structure. While consensus was not achieved on the diagnostic algorithm, this reflects wider debate in the field concerning the presence and control of background pain and the distinction between breakthrough, episodic, and end-of-dose pain types. The present study supports the conceptual foundation of the BTPAQ-SR and justifies its continued development. The next phase will evaluate the tool’s comprehensibility, comprehensiveness, clarity, and acceptability through cognitive interviews with children and young people. This will be followed by further modifications and rigorous psychometric testing to establish internal consistency, test–retest reliability, construct validity, convergent validity, discriminant validity, and responsiveness.

## Figures and Tables

**Table 1:** Socio-demographic and Clinical Experience Characteristics of Participants (n = 53).

Baseline Characteristic	Number	Percentage (%)
Gender		
Female	46	92
Male	4	8
Ethnic Background		
Indian	1	2
Pakistani	1	2
Chinese	1	2
Any other Asian background	1	2
African	1	2
White and Asian	1	2
English, Welsh, Scottish, Northern Irish or British	27	54
Irish	3	6
Any other White background	13	26
Any other ethnic group	1	2
Profession		
Physician	26	52
Nurse	21	42
Psychologist	1	2
Physiotherapist	1	2
Other—Clinical officer	1	2
Primary Role		
Clinician	46	92
Researcher	1	2
Other—Nurse manager	1	2
Other—Nurse specialist	1	2
Other—Team Lead and CNS	1	2
Country of Practice		
United Kingdom	38	76
Europe—Sweden	1	2
USA	3	6
Canada	3	6
Australia	2	4
New Zealand	1	2
Other—Latin America, Panama	1	2
Other—Malawi	1	2
Years of Experience in Pain Management		
3–5 years	6	12
6–10 years	10	20
> 10 years	34	68
Number of patients (8–25 yrs) with LLC or LTC seen per year		
<10	5	10
10–50	18	36
51–100	11	22
>100	16	32
Number of patients (8–25 yrs) with LLC or LTC per year with BTP		
<25%	8	16
25–50%	20	40
51–75%	11	22
>75%	11	22

**Table 2:** Content Analysis Framework for e-Delphi Survey Open-Text Responses on the BTPAQ-SR.

Theme	Category	Subcategory
A Controversial Diagnostic Algorithm: Challenges and Considerations	Algorithm usefulness (n = 8)	Easy to understand (n = 7)
		Particularly helpful for less experienced healthcare professionals (n = 1)
	Diagnosis of Breakthrough Pain (BTP) Based on Background Pain (n = 9)	Aids in differentiating BTP from background pain (n = 3)
		Concerns about diagnosing BTP solely based on background pain (n = 6)
	Questions and Concerns About the Algorithm (n = 10)	Difficulty differentiating between types of pain (n = 2)
		Ambivalence around the use of the “12 h” criterion (n = 6)
		Algorithm is limited when used in isolation (n = 2)
		Ambiguity around the term “brief” pain increases (n = 2)
	Suggestions for Improving the Algorithm (n = 6)	
BTPAQ-SR: A Valuable Assessment Tool in Need of Refinement	Benefits and Value of the BTPAQ-SR (n = 10)	Supports individualised assessment (n = 5)
		Clinical utility (n = 5)
	Descriptors of Breakthrough Pain (BTP) (n = 14)	
	Suitability and relevance of activity-related items (n = 5)	
	Impact of BTP (n = 3)	
	Structure and format of the BTPAQ-SR (n = 10)	Wording clarity and appropriateness (n = 3)
		Overall structure and organisation (n = 1)
		Response format (n = 1)
		Inclusion and value of the free-text box (n = 4)
		Usefulness of the numerical pain rating scale (n = 1)
Key Considerations in the Assessment of Breakthrough Pain and the Role of the BTPAQ-SR	Importance of identifying the type of breakthrough pain (BTP) (n = 4)	Distinguishing between different types of pain is essential (n = 1)
	Significance of clearly defining and assessing BTP (n = 3)	BTP assessment is often complex and nuanced (n = 3)
	Clarifying the purpose and aim of the BTPAQ-SR (n = 2)	
	Similarities in wording with other existing pain assessment tools (n = 2)	

Note: n = number of participants.

## Data Availability

The raw data supporting the conclusions of this article will be made available by the authors upon reasonable request.

## Abbreviations

The following abbreviations are used in this manuscript:

BTP	breakthrough pain
BTPAQ	Breakthrough Pain Assessment Questionnaire
BTPAQ-SR	Breakthrough Pain Assessment Questionnaire-self-report version
LLC/LTC	life-limiting and life-threatening conditions
PROMs	patient-reported outcome measures

## Appendix A

**Table A1:** The Mode and Interquartile Range (IQR) of Individual Item Consensus in Delphi Round 1 (n = 50).

Item	Importance		Frequency	
	Mode	IQR	Mode	IQR
Algorithm	2	2		
1. Body Map	3	2		
2. The following bring on my BTP:				
2a. Movement (e.g., walking, playing active games)	4	1	4	1
2b. Changing position (e.g., turning sides on my bed)	4	1	4	1
2c. Actions/medical procedures that are done for my illness (e.g., changing a dressing or cannula)	4	1	4	1
2d. Medications	3	2	2	1
2e. Eating and/or drinking	3	1	3	1
2f. Needing another dose of my normal pain medication	4	1	3	2
2g. Anything else	4	2	3	1
3. I have BTP even when I am still (e.g., when I am standing, sitting or lying still)	5	1	3	1
4. How bad is your BTP generally?	5	1		
5. How bad is the worst BTP you experience?	4	1		
6. From the time you first notice your BTP how long does it take to get really bad?	3	1		
7. How does your BTP usually start?	3	1		
8. How often does your BTP happen?	4	1		
9. How long does your BTP usually last?	4	1		
10. My BTP feels like:				
10a. Aching	3	2	3	1
10b. Throbbing	3	1	3	0
10c. Squeezing	3	1	3	0
10d. Gnawing	3	1	3	1
10e. Crampy	3	2	3	1
10f. Burning	4	2	3	1
10g. Sharp	3	2	3	1
10h. Stabbing	3	1	3	1
10i. Crushing	3	1	3	1
10j. Shooting	4	1	3	1
10k. Splitting	3	1	2	2
10l. Heavy Pressure	3	1	3	1
10m. Pins and Needles	4	2	3	1
10n. Spasms	3	1	3	1
10o. Stiffness	3	1	3	1
10p. Unsure/can’t tell	3	2	3	1
10q. Other	4	2		
11. Please write down what makes your BTP better	5	1		
12. Please write down what makes your BTP worse	5	1		
13. I worry about my BTP	4	1	4	1
14. I feel sad about my BTP	5	2	4	1
15. I feel scared about my BYP	4	2	3	1
16. Nothing is fun anymore because of my BTP	5	2	3	1
17. My BTP stops me from doing the following:				
17a. Enjoying time with family	4	1	3	1
17b. Enjoying time with friends	5	2	3	1
17c. Going to school/college/work	4	1	4	1
17d. Playing/participating in recreational activities	4	2	3	1
17e. Sleeping	5	1	4	0
17f. Walking	4	2	3	1
17g. Exercising	3	1	3	1
17h. Doing daily activities like getting dressed	4	2	3	1
17i. Other, please write it down here	3	1	3	0
18. Please write down below anything else you want to say about BTP	5	2		

## Appendix B

**Table A2:** The Number (%) of Individual Item Consensus and Grouped Consensus (3–5) for Delphi Round 1 (n = 50).

Item	Importance												Frequency											
	Not at all		Somewhat		Important		Very Important		Extremely Important		Total consensus (3–5)		Never/Almost never		Rarely		Sometimes		Often		Always/Almost always		Total consensus (3–5)	
	*n*	%	*n*	%	*n*	%	*n*	%	*n*	%	***n***	**%**	*n*	%	*n*	%	*n*	%	*n*	%	*n*	%	***n***	**%**
**Algorithm**	3	6	20	40	13	26	10	20	4	8	**27**	**54**	N/A	N/A	N/A	N/A	N/A	N/A	N/A	N/A	N/A	N/A	**N/A**	**N/A**
**1. Body Map**	0	0	6	12	16	32	15	30	13	26	**44**	**88**	N/A	N/A	N/A	N/A	N/A	N/A	N/A	N/A	N/A	N/A	**N/A**	**N/A**
**2. The following bring on my BTP:**																								
**2a. Movement**	0	0	0	0	12	24	22	44	16	33	**50**	**100**	1	2	0	0	20	40	25	50	4	8	**49**	**98**
**2b. Changing Position**	0	0	1	2	17	34	20	40	12	24	**49**	**98**	0	0	0	0	20	40	24	48	6	12	**50**	**100**
**2c. Actions/Medical Procedures**	2	4	8	16	10	20	18	36	12	24	**40**	**80**	1	2	3	6	20	40	21	42	5	10	**46**	**92**
**2d. Medications**	2	4	14	28	15	30	12	24	7	14	**34**	**68**	11	22	23	46	13	26	0	0	3	6	**16**	**32**
**2e. Eating/Drinking**	2	4	6	12	17	34	17	34	8	16	**42**	**84**	2	4	13	26	28	56	7	14	0	0	**35**	**70**
**2f. Medication Dose**	4	8	6	12	12	24	17	34	11	22	**40**	**80**	7	14	9	18	19	38	12	24	3	6	**34**	**68**
**2g. Anything else**	0	0	3	6	15	30	19	38	13	26	**47**	**94**	0	0	6	12	25	50	16	32	3	6	**44**	**88**
**3. BTP when still**	0	0	1	2	6	12	21	42	22	44	**49**	**98**	0	0	5	10	24	48	20	40	1	2	**45**	**90**
**4. BTP generally**	0	0	4	8	8	16	17	34	21	42	**46**	**92**	N/A	N/A	N/A	N/A	N/A	N/A	N/A	N/A	N/A	N/A	**N/A**	**N/A**
**5. BTP worst**	0	0	4	8	6	12	23	46	17	34	**46**	**92**	N/A	N/A	N/A	N/A	N/A	N/A	N/A	N/A	N/A	N/A	**N/A**	**N/A**
**6. BTP length**	0	0	7	14	17	34	14	28	12	24	**43**	**86**	N/A	N/A	N/A	N/A	N/A	N/A	N/A	N/A	N/A	N/A	**N/A**	**N/A**
**7. BTP usually start**	1	2	7	14	17	34	14	28	11	22	**42**	**84**	N/A	N/A	N/A	N/A	N/A	N/A	N/A	N/A	N/A	N/A	**N/A**	**N/A**
**8. BTP how often**	0	0	1	2	9	18	20	40	20	40	**49**	**98**	N/A	N/A	N/A	N/A	N/A	N/A	N/A	N/A	N/A	N/A	**N/A**	**N/A**
**9. How long**	1	2	1	2	10	20	23	46	15	30	**48**	**96**	N/A	N/A	N/A	N/A	N/A	N/A	N/A	N/A	N/A	N/A	**N/A**	**N/A**
**10. My BTP feels like:**																								
**10a. Aching**	1	2	4	8	18	36	12	24	15	30	**45**	**90**	0	0	5	10	27	54	17	34	1	2	**45**	**90**
**10b. Throbbing**	0	0	8	16	19	38	12	24	11	22	**42**	**84**	2	4	5	10	33	66	10	20	0	0	**43**	**86**
**10c. Squeezing**	1	2	5	10	22	44	10	20	12	24	**44**	**88**	0	0	10	20	35	70	5	10	0	0	**40**	**80**
**10d. Gnawing**	2	4	8	16	21	42	9	18	10	20	**40**	**80**	5	10	15	30	25	50	4	8	1	2	**30**	**60**
**10e. Crampy**	0	0	3	6	22	44	12	24	13	26	**47**	**94**	1	2	4	8	29	58	15	30	1	2	**45**	**90**
**10f. Burning**	0	0	2	4	15	30	19	38	14	28	**48**	**96**	0	0	1	2	31	62	16	32	2	4	**49**	**98**
**10g. Sharp**	0	0	2	4	18	36	17	34	13	26	**48**	**96**	0	0	1	2	23	46	21	42	5	10	**49**	**98**
**10h. Stabbing**	0	0	3	6	21	42	14	28	12	24	**47**	**94**	0	0	3	6	22	44	22	44	3	6	**47**	**94**
**10i. Crushing**	0	0	10	20	19	38	10	20	11	22	**40**	**80**	5	10	17	34	25	50	2	4	1	2	**28**	**56**
**10j. Shooting**	1	2	2	4	17	34	18	36	12	24	**47**	**94**	2	4	5	10	23	46	18	36	2	4	**43**	**86**
**10k. Splitting**	5	10	13	26	20	40	4	8	8	16	**32**	**64**	14	28	19	38	16	32	1	2	0	0	**17**	**34**
**10l. Heavy Pressure**	2	4	8	16	23	46	8	16	9	18	**40**	**80**	4	8	19	38	25	50	1	2	1	2	**27**	**54**
**10m. Pins and Needles**	0	0	3	6	16	32	18	36	13	26	**47**	**94**	0	0	2	4	30	60	17	34	1	2	**48**	**96**
**10n. Spasms**	0	0	3	6	19	38	17	34	11	22	**47**	**94**	0	0	5	10	26	52	18	36	1	2	**45**	**90**
**10o. Stiffness**	0	0	11	22	18	36	12	24	9	18	**39**	**78**	4	8	10	20	27	54	8	16	1	2	**36**	**72**
**10p. Unsure**	0	0	7	14	15	30	14	28	14	28	**43**	**86**	0	0	5	10	22	44	20	40	3	6	**45**	**90**
**10q. Other**	2	4	5	10	14	28	16	32	13	26	**43**	**86**	N/A	N/A	N/A	N/A	N/A	N/A	N/A	N/A	N/A	N/A	**N/A**	**N/A**
**11. BTP Better**	0	0	0	0	4	8	21	42	25	50	**50**	**100**	N/A	N/A	N/A	N/A	N/A	N/A	N/A	N/A	N/A	N/A	**N/A**	**N/A**
**12. BTP Worse**	0	0	0	0	4	8	21	42	25	50	**50**	**100**	N/A	N/A	N/A	N/A	N/A	N/A	N/A	N/A	N/A	N/A	**N/A**	**N/A**
**13. BTP Worry**	0	0	3	6	8	16	22	44	17	34	**47**	**94**	0	0	2	4	21	42	22	44	5	10	**48**	**96**
**14. BTP Sad**	0	0	6	12	13	26	14	28	17	34	**44**	**88**	1	2	6	12	20	40	21	42	2	4	**43**	**86**
**15. BTP Scared**	0	0	2	4	11	22	19	38	18	36	**48**	**96**	0	0	3	6	22	44	21	42	4	8	**47**	**94**
**16. BTP Nothing fun**	0	0	3	6	10	20	18	36	19	38	**47**	**94**	0	0	4	8	28	56	16	32	2	4	**46**	**92**
**17. My BTP stops me from doing the following:**																								
**17a. Family Time**	0	0	2	4	10	20	19	38	19	38	**48**	**96**	0	0	8	16	21	42	20	40	1	2	**42**	**84**
**17b. Friends Time**	0	0	2	4	11	22	18	36	19	38	**48**	**98**	0	0	5	10	24	48	19	38	2	4	**45**	**90**
**17c. School**	1	2	1	2	7	14	24	48	18	34	**49**	**96**	1	2	2	4	19	38	24	48	4	8	**47**	**94**
**17d. Activities**	0	0	4	8	10	20	21	42	15	30	**46**	**92**	0	0	1	2	26	52	22	44	1	2	**49**	**98**
**17e. Sleeping**	0	0	1	2	3	6	20	40	26	52	**49**	**98**	0	0	0	0	12	24	36	72	2	4	**50**	**100**
**17f. Walking**	0	0	1	2	16	32	18	36	15	30	**49**	**98**	1	2	2	4	25	50	20	40	2	4	**47**	**94**
**17g. Exercising**	1	2	11	22	14	28	13	26	11	22	**38**	**76**	3	6	6	12	22	44	14	28	5	10	**41**	**82**
**17h Daily Activities**	0	0	1	2	14	28	19	38	16	32	**49**	**98**	0	0	5	10	27	54	14	28	4	8	**45**	**90**
**17i Other**	2	4	4	6	17	34	17	34	11	22	**45**	**90**	4	8	1	2	34	68	8	16	3	6	**45**	**90**
**18 Additional Comments**	1	2	3	6	12	24	14	28	20	40	**46**	**92**	N/A	N/A	N/A	N/A	N/A	N/A	N/A	N/A	N/A	N/A	**N/A**	**N/A**
